# 2-(Mesitylmethylsulfanyl)pyridine *N*-oxide monohydrate

**DOI:** 10.1107/S1600536808029255

**Published:** 2008-09-17

**Authors:** B. Ravindran Durai Nayagam, Samuel Robinson Jebas, H. Johnson Jeyakumar, Dieter Schollmeyer

**Affiliations:** a Department of Chemistry, Popes College, Sawyerpuram 628251, Tamilnadu, India; b Department of Physics, Karunya University, Karunya Nagar, Coimbatore 64114, India; c Department of Physics, Popes College, Sawyerpuram 628251, Tamilnadu, India; d Institut für Organische Chemie, Universität Mainz, Duesbergweg 10-14, 55099 Mainz, Germany

## Abstract

In the title compound, C_15_H_17_NOS·H_2_O, the benzene and pyridine rings form a dihedral angle of 71.18 (2)°. The intra­molecular S⋯O distance [2.737 (3) Å] is shorter than expected and, in terms of hybridization principles, the N—C—S angle [114.1 (2)°] is smaller than expected. The crystal structure is stabilized by inter­molecular O—H⋯O and weak C—H⋯O hydrogen bonds. In addition, weak π–π stacking inter­actions with a centroid–centroid distance of 3.778 (3) Å are also observed.

## Related literature

For related structures, see: Jebas *et al.* (2005 ▶); Hartung *et al.* (1996 ▶); Ravindran Durai Nayagam *et al.* (2008 ▶). For biological activities of *N*-oxide derivatives, see: Bovin *et al.* (1992 ▶); Katsuyuki *et al.* (1991 ▶); Leonard *et al.* (1955 ▶); Lobana & Bhatia (1989 ▶); Symons & West (1985 ▶). For bond-length data, see: Allen *et al.* (1987 ▶).
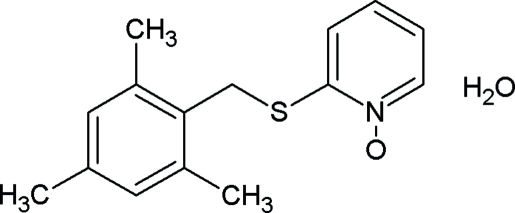


## Experimental

### 

#### Crystal data

C_15_H_17_NOS·H_2_O
*M* *_r_* = 277.37
Monoclinic, 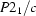

*a* = 12.358 (7) Å
*b* = 15.404 (6) Å
*c* = 7.748 (5) Å
β = 106.40 (2)°
*V* = 1415.0 (13) Å^3^
*Z* = 4
Cu *K*α radiation
μ = 2.01 mm^−1^
*T* = 193 (2) K
0.50 × 0.20 × 0.05 mm

#### Data collection

Enraf–Nonius CAD-4 diffractometer
Absorption correction: ψ scan (*CORINC*; Dräger & Gattow, 1971 ▶) *T*_min_ = 0.67, *T*_max_ = 0.99 (expected range = 0.612–0.904)
2896 measured reflections
2684 independent reflections
2048 reflections with *I* > 2σ(*I*)
*R*_int_ = 0.067
3 standard reflections frequency: 60 min intensity decay: 3%

#### Refinement

*R*[*F* ^2^ > 2σ(*F* ^2^)] = 0.067
*wR*(*F* ^2^) = 0.190
*S* = 1.06
2684 reflections
175 parameters
H-atom parameters constrained
Δρ_max_ = 0.57 e Å^−3^
Δρ_min_ = −0.79 e Å^−3^

### 

Data collection: *CAD-4 EXPRESS* (Enraf–Nonius, 1994 ▶); cell refinement: *CAD-4 EXPRESS*; data reduction: *CORINC* (Dräger & Gattow, 1971 ▶); program(s) used to solve structure: *SHELXS97* (Sheldrick, 2008 ▶); program(s) used to refine structure: *SHELXL97* (Sheldrick, 2008 ▶); molecular graphics: *SHELXTL* (Sheldrick, 2008 ▶); software used to prepare material for publication: *SHELXTL* and *PLATON* (Spek, 2003 ▶).

## Supplementary Material

Crystal structure: contains datablocks global, I. DOI: 10.1107/S1600536808029255/lh2692sup1.cif

Structure factors: contains datablocks I. DOI: 10.1107/S1600536808029255/lh2692Isup2.hkl

Additional supplementary materials:  crystallographic information; 3D view; checkCIF report

## Figures and Tables

**Table 1:** Hydrogen-bond geometry (Å, °)

*D*—H⋯*A*	*D*—H	H⋯*A*	*D*⋯*A*	*D*—H⋯*A*
O1*W*—H1*W*⋯O18	0.84	2.05	2.875 (4)	165
O1*W*—H2*W*⋯O18^i^	0.84	2.17	2.869 (4)	141
C16—H16⋯O1*W*	0.95	2.58	3.226 (6)	125

Symmetry code: (i) 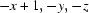.

**Fig. 1.**
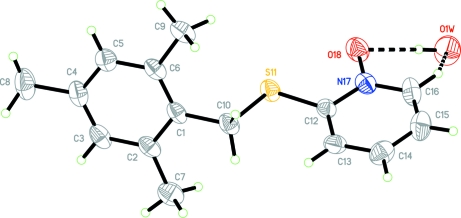
The molecular structure of the title compound, showing 50% probability displacement ellipsoids and the atom numbering scheme. Hydrogen bonds are shown as dashed lines.

**Fig. 2.**
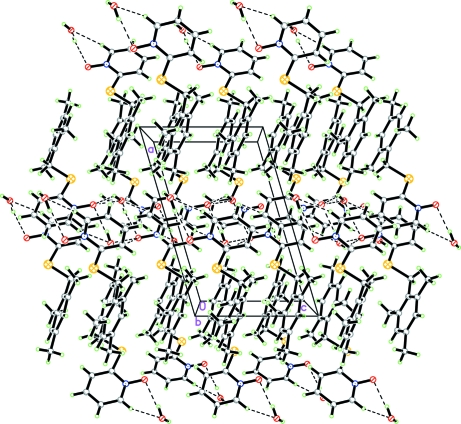
Part of the crystal structure of the title compound, viewed along the b axis showing hydrogen bonds as dashed lines.

## supplementary crystallographic information

### Comment

N-oxides and their derivatives show a broad spectrum of biological activity such as antifungal, antimicrobial and antibacterial activities (Lobana & Bhatia, 1989; Symons *et al.*, 1985). These compounds are also found to be involved in DNA strand scission under physiological conditions (Katsuyuki *et al.*, 1991; Bovin *et al.*, 1992). Pyridine N-oxides bearing a sulfur group in the 2 position display significant antimicrobial activity (Leonard *et al.*, 1955). In view of the importance of N-oxides, we have previously reported the crystal structures of N–oxide derivatives (Jebas *et al.*, 2005; Ravindran Durai Nayagam, *et al.*, 2008). As an extension of our work, we report here the crystal structure of the title compound.

The asymmetric unit of (I), consists of one molecule 2-(1-oxo-2-pyridylsulfanylmethyl) mesitylene and a water molecule. The bond lengths and angles agree well with the N-oxide derivatives reported earlier (Jebas *et al.*, 2005; Ravindran Durai Nayagam *et al.*, 2008). The N—O bond length is in good agreement with the mean value of 1.304 (15) Å reported in the literature for pyridine N-oxides (Allen *et al.*, 1987).

The pyridine ring and the benzene rings are essentially individually planar with the maximum deviation from planarity being 0.011 (2) Å for atom C2 and -0.010 (2) Å for atom C12 respectively. The dihedral angle formed by the benzene ring (C1–C6) and the pyridine ring (C12–C16/N17) is 71.18 (2)°. The atom O18 attached to atom N17 of the pyridine ring is essentially co-planar; the relevant torsion angle being O18—N17—C16—C15 = 178.9 (3)°.

The crystal structure is stabilized by intermolecular O—H···O and C—H···O hydrogen bonds. In addition, π–π interactions with Cg1···Cg1^i^ = 3.778 (3) Å (*Cg*1 is the centroid defined by ring atoms C12–C16/N17) [symmetry code:(i) 1-*x*,-y,1-*z*] are observed. As in the structure of 2-(1-phenyl-4-penten-l-yl-thio)pyridine N-oxide (Hartung *et al.*, 1996) a short intramolecular S···O [2.737 (3) Å] distance is observed.

### Experimental

A mixture of mono(bromomethyl)mesitylene (0.213 g, 1 mmol) and 1-hydroxypyridine-2-thione sodium salt (0.149,1 mmol) in water (30 ml) and methanol (30 ml) was heated at 333 K with stirring for 30 min. The compound formed was filtered off, and dried. The compound was dissolved in acetone and water (1: 1v/v) and allowed to undergo slow evaporation. Colourless crystals were obtained after a week

### Refinement

After checking for their presence in the Fourier map, all the hydrogen atoms were placed in calculated positions and allowed to ride on their parent atoms with the C—H = 0.95 Å (aromatic); C—H = 0.99 Å(methylene); C—H = 0.98 Å (methyl) and O—H = 0.84 Å with *U*_iso_(H) in the range of 1.2U_eq_(C)–1.5U_eq_(C,O)methyl.

### Crystal data

**Table:**

C_15_H_17_NOS·H_2_O	*F*(000) = 592
*M**_r_* = 277.37	*D*_x_ = 1.302 Mg m^−^^3^
Monoclinic, *P*2_1_/*c*	Cu *K*α radiation, λ = 1.54178 Å
Hall symbol: -P 2ybc	Cell parameters from 25 reflections
*a* = 12.358 (7) Å	θ = 36–50°
*b* = 15.404 (6) Å	µ = 2.01 mm^−^^1^
*c* = 7.748 (5) Å	*T* = 193 K
β = 106.40 (2)°	Plate, colourless
*V* = 1415.0 (13) Å^3^	0.50 × 0.20 × 0.05 mm
*Z* = 4	

### Data collection

**Table:**

Enraf–Nonius CAD-4 diffractometer	2048 reflections with *I* > 2σ(*I*)
Radiation source: rotating anode	*R*_int_ = 0.067
graphite	θ_max_ = 70.0°, θ_min_ = 3.7°
ω/2θ scans	*h* = −14→15
Absorption correction: ψ scan (CORINC; Dräger & Gattow, 1971)	*k* = −18→0
*T*_min_ = 0.67, *T*_max_ = 0.99	*l* = −9→0
2896 measured reflections	3 standard reflections every 60 min
2684 independent reflections	intensity decay: 3%

### Refinement

**Table:**

Refinement on *F*^2^	Primary atom site location: structure-invariant direct methods
Least-squares matrix: full	Secondary atom site location: difference Fourier map
*R*[*F*^2^ > 2σ(*F*^2^)] = 0.067	Hydrogen site location: inferred from neighbouring sites
*wR*(*F*^2^) = 0.190	H-atom parameters constrained
*S* = 1.06	*w* = 1/[σ^2^(*F*_o_^2^) + (0.1179*P*)^2^ + 0.1489*P*] where *P* = (*F*_o_^2^ + 2*F*_c_^2^)/3
2684 reflections	(Δ/σ)_max_ < 0.001
175 parameters	Δρ_max_ = 0.58 e Å^−^^3^
0 restraints	Δρ_min_ = −0.79 e Å^−^^3^

### Special details

**Table:**

Geometry. All e.s.d.'s (except the e.s.d. in the dihedral angle between two l.s. planes) are estimated using the full covariance matrix. The cell e.s.d.'s are taken into account individually in the estimation of e.s.d.'s in distances, angles and torsion angles; correlations between e.s.d.'s in cell parameters are only used when they are defined by crystal symmetry. An approximate (isotropic) treatment of cell e.s.d.'s is used for estimating e.s.d.'s involving l.s. planes.
Refinement. Refinement of *F*^2^ against ALL reflections. The weighted *R*-factor *wR* and goodness of fit *S* are based on *F*^2^, conventional *R*-factors *R* are based on *F*, with *F* set to zero for negative *F*^2^. The threshold expression of *F*^2^ > σ(*F*^2^) is used only for calculating *R*-factors(gt) *etc*. and is not relevant to the choice of reflections for refinement. *R*-factors based on *F*^2^ are statistically about twice as large as those based on *F*, and *R*- factors based on ALL data will be even larger.

### Fractional atomic coordinates and isotropic or equivalent isotropic displacement parameters (Å^2^)

**Table:**

	*x*	*y*	*z*	*U*_iso_*/*U*_eq_	
C1	0.0724 (3)	−0.0867 (2)	0.3741 (4)	0.0305 (7)	
C2	0.0757 (3)	−0.1748 (2)	0.4252 (4)	0.0336 (7)	
C3	−0.0205 (3)	−0.2249 (2)	0.3573 (4)	0.0375 (8)	
H3	−0.0199	−0.2837	0.3946	0.045*	
C4	−0.1170 (3)	−0.1924 (2)	0.2377 (4)	0.0376 (8)	
C5	−0.1186 (3)	−0.1053 (2)	0.1862 (4)	0.0355 (7)	
H5	−0.1844	−0.0818	0.1045	0.043*	
C6	−0.0247 (3)	−0.05268 (19)	0.2537 (4)	0.0314 (7)	
C7	0.1771 (3)	−0.2149 (2)	0.5531 (5)	0.0469 (9)	
H7A	0.1954	−0.1835	0.6678	0.070*	
H7B	0.2411	−0.2117	0.5022	0.070*	
H7C	0.1612	−0.2758	0.5733	0.070*	
C8	−0.2178 (4)	−0.2507 (3)	0.1647 (6)	0.0561 (11)	
H8A	−0.2837	−0.2153	0.1054	0.084*	
H8B	−0.2332	−0.2833	0.2637	0.084*	
H8C	−0.2020	−0.2913	0.0775	0.084*	
C9	−0.0327 (3)	0.0412 (2)	0.1961 (5)	0.0379 (8)	
H9A	0.0247	0.0534	0.1341	0.057*	
H9B	−0.0202	0.0786	0.3022	0.057*	
H9C	−0.1078	0.0526	0.1144	0.057*	
C10	0.1718 (3)	−0.0290 (2)	0.4538 (4)	0.0354 (7)	
H10A	0.2203	−0.0556	0.5653	0.042*	
H10B	0.1454	0.0280	0.4850	0.042*	
S11	0.25245 (7)	−0.01425 (5)	0.29196 (10)	0.0343 (3)	
C12	0.3569 (3)	0.05537 (19)	0.4133 (4)	0.0300 (7)	
C13	0.3783 (3)	0.0803 (2)	0.5928 (4)	0.0364 (7)	
H13	0.3343	0.0567	0.6640	0.044*	
C14	0.4623 (3)	0.1386 (2)	0.6672 (5)	0.0454 (9)	
H14	0.4765	0.1552	0.7897	0.054*	
C15	0.5265 (3)	0.1733 (2)	0.5633 (6)	0.0487 (9)	
H15	0.5839	0.2147	0.6128	0.058*	
C16	0.5057 (3)	0.1469 (2)	0.3886 (6)	0.0446 (9)	
H16	0.5501	0.1697	0.3171	0.053*	
N17	0.4236 (2)	0.08942 (17)	0.3157 (4)	0.0336 (6)	
O18	0.4037 (2)	0.06592 (17)	0.1461 (3)	0.0446 (6)	
O1W	0.6227 (3)	0.0815 (2)	0.0886 (4)	0.0610 (8)	
H1W	0.5575	0.0690	0.0933	0.091*	
H2W	0.6495	0.0394	0.0452	0.091*	

### Atomic displacement parameters (Å^2^)

**Table:**

	*U*^11^	*U*^22^	*U*^33^	*U*^12^	*U*^13^	*U*^23^
C1	0.0458 (18)	0.0265 (14)	0.0256 (14)	−0.0078 (13)	0.0208 (14)	−0.0057 (11)
C2	0.0497 (19)	0.0286 (16)	0.0288 (15)	−0.0017 (14)	0.0214 (14)	−0.0011 (13)
C3	0.057 (2)	0.0265 (15)	0.0356 (17)	−0.0102 (14)	0.0246 (16)	−0.0070 (13)
C4	0.0480 (19)	0.0392 (17)	0.0332 (16)	−0.0162 (15)	0.0239 (15)	−0.0141 (14)
C5	0.0420 (18)	0.0388 (18)	0.0300 (16)	−0.0035 (14)	0.0171 (14)	−0.0055 (13)
C6	0.0492 (19)	0.0263 (15)	0.0255 (14)	−0.0026 (13)	0.0217 (14)	−0.0050 (12)
C7	0.058 (2)	0.0377 (19)	0.048 (2)	0.0032 (17)	0.0194 (18)	0.0037 (16)
C8	0.062 (3)	0.058 (2)	0.055 (2)	−0.030 (2)	0.027 (2)	−0.020 (2)
C9	0.060 (2)	0.0264 (16)	0.0334 (16)	0.0007 (14)	0.0230 (16)	−0.0007 (12)
C10	0.0476 (19)	0.0363 (16)	0.0261 (15)	−0.0109 (14)	0.0167 (14)	−0.0030 (13)
S11	0.0424 (5)	0.0364 (5)	0.0281 (4)	−0.0087 (3)	0.0164 (3)	−0.0048 (3)
C12	0.0309 (15)	0.0228 (14)	0.0361 (16)	0.0022 (12)	0.0091 (13)	0.0048 (12)
C13	0.0443 (18)	0.0318 (16)	0.0312 (16)	0.0004 (14)	0.0075 (14)	0.0038 (13)
C14	0.046 (2)	0.0403 (19)	0.045 (2)	0.0026 (16)	0.0045 (17)	−0.0067 (16)
C15	0.0396 (19)	0.039 (2)	0.068 (3)	−0.0066 (15)	0.0159 (18)	−0.0110 (18)
C16	0.0367 (18)	0.0357 (18)	0.066 (2)	−0.0061 (15)	0.0230 (18)	−0.0009 (17)
N17	0.0343 (14)	0.0293 (13)	0.0402 (15)	0.0044 (11)	0.0154 (12)	0.0042 (11)
O18	0.0475 (15)	0.0501 (15)	0.0417 (14)	−0.0042 (11)	0.0213 (12)	0.0012 (11)
O1W	0.0589 (18)	0.085 (2)	0.0441 (15)	−0.0134 (16)	0.0233 (14)	−0.0132 (15)

### Geometric parameters (Å, °)

**Table:**

C1—C6	1.397 (5)	C9—H9B	0.9800
C1—C2	1.411 (4)	C9—H9C	0.9800
C1—C10	1.500 (4)	C10—S11	1.823 (3)
C2—C3	1.390 (5)	C10—H10A	0.9900
C2—C7	1.494 (5)	C10—H10B	0.9900
C3—C4	1.381 (5)	S11—C12	1.736 (3)
C3—H3	0.9500	C12—N17	1.371 (4)
C4—C5	1.399 (5)	C12—C13	1.394 (4)
C4—C8	1.509 (5)	C13—C14	1.371 (5)
C5—C6	1.391 (5)	C13—H13	0.9500
C5—H5	0.9500	C14—C15	1.388 (6)
C6—C9	1.509 (4)	C14—H14	0.9500
C7—H7A	0.9800	C15—C16	1.367 (6)
C7—H7B	0.9800	C15—H15	0.9500
C7—H7C	0.9800	C16—N17	1.345 (4)
C8—H8A	0.9800	C16—H16	0.9500
C8—H8B	0.9800	N17—O18	1.318 (4)
C8—H8C	0.9800	O1W—H1W	0.8400
C9—H9A	0.9800	O1W—H2W	0.8400
			
C6—C1—C2	120.0 (3)	C6—C9—H9B	109.5
C6—C1—C10	120.1 (3)	H9A—C9—H9B	109.5
C2—C1—C10	119.9 (3)	C6—C9—H9C	109.5
C3—C2—C1	118.3 (3)	H9A—C9—H9C	109.5
C3—C2—C7	119.3 (3)	H9B—C9—H9C	109.5
C1—C2—C7	122.4 (3)	C1—C10—S11	109.5 (2)
C4—C3—C2	122.5 (3)	C1—C10—H10A	109.8
C4—C3—H3	118.7	S11—C10—H10A	109.8
C2—C3—H3	118.7	C1—C10—H10B	109.8
C3—C4—C5	118.6 (3)	S11—C10—H10B	109.8
C3—C4—C8	120.1 (3)	H10A—C10—H10B	108.2
C5—C4—C8	121.3 (4)	C12—S11—C10	99.89 (16)
C6—C5—C4	120.6 (3)	N17—C12—C13	118.1 (3)
C6—C5—H5	119.7	N17—C12—S11	114.1 (2)
C4—C5—H5	119.7	C13—C12—S11	127.8 (3)
C5—C6—C1	120.0 (3)	C14—C13—C12	120.5 (3)
C5—C6—C9	118.0 (3)	C14—C13—H13	119.7
C1—C6—C9	122.0 (3)	C12—C13—H13	119.7
C2—C7—H7A	109.5	C13—C14—C15	119.9 (4)
C2—C7—H7B	109.5	C13—C14—H14	120.1
H7A—C7—H7B	109.5	C15—C14—H14	120.1
C2—C7—H7C	109.5	C16—C15—C14	118.8 (3)
H7A—C7—H7C	109.5	C16—C15—H15	120.6
H7B—C7—H7C	109.5	C14—C15—H15	120.6
C4—C8—H8A	109.5	N17—C16—C15	121.4 (3)
C4—C8—H8B	109.5	N17—C16—H16	119.3
H8A—C8—H8B	109.5	C15—C16—H16	119.3
C4—C8—H8C	109.5	O18—N17—C16	120.4 (3)
H8A—C8—H8C	109.5	O18—N17—C12	118.2 (3)
H8B—C8—H8C	109.5	C16—N17—C12	121.3 (3)
C6—C9—H9A	109.5	H1W—O1W—H2W	109.5
			
C6—C1—C2—C3	−2.0 (4)	C6—C1—C10—S11	−80.1 (3)
C10—C1—C2—C3	176.0 (3)	C2—C1—C10—S11	101.9 (3)
C6—C1—C2—C7	179.9 (3)	C1—C10—S11—C12	178.5 (2)
C10—C1—C2—C7	−2.1 (5)	C10—S11—C12—N17	−170.9 (2)
C1—C2—C3—C4	2.3 (5)	C10—S11—C12—C13	8.2 (3)
C7—C2—C3—C4	−179.6 (3)	N17—C12—C13—C14	1.3 (5)
C2—C3—C4—C5	−1.5 (5)	S11—C12—C13—C14	−177.7 (3)
C2—C3—C4—C8	178.3 (3)	C12—C13—C14—C15	0.2 (5)
C3—C4—C5—C6	0.3 (5)	C13—C14—C15—C16	−1.3 (6)
C8—C4—C5—C6	−179.5 (3)	C14—C15—C16—N17	1.0 (6)
C4—C5—C6—C1	0.0 (4)	C15—C16—N17—O18	178.9 (3)
C4—C5—C6—C9	−178.7 (3)	C15—C16—N17—C12	0.5 (5)
C2—C1—C6—C5	0.9 (4)	C13—C12—N17—O18	179.9 (3)
C10—C1—C6—C5	−177.1 (3)	S11—C12—N17—O18	−0.9 (4)
C2—C1—C6—C9	179.5 (3)	C13—C12—N17—C16	−1.7 (5)
C10—C1—C6—C9	1.5 (4)	S11—C12—N17—C16	177.5 (3)

### Hydrogen-bond geometry (Å, °)

**Table:**

*D*—H···*A*	*D*—H	H···*A*	*D*···*A*	*D*—H···*A*
O1W—H1W···O18	0.84	2.05	2.875 (4)	165.
O1W—H2W···O18^i^	0.84	2.17	2.869 (4)	141.
C16—H16···O1W	0.95	2.58	3.226 (6)	125

Symmetry codes: (i) −*x*+1, −*y*, −*z*.
